# IL-22 Protects against Tissue Damage during Cutaneous Leishmaniasis

**DOI:** 10.1371/journal.pone.0134698

**Published:** 2015-08-18

**Authors:** Ciara Gimblet, Michael A. Loesche, Lucas Carvalho, Edgar M. Carvalho, Elizabeth A. Grice, David Artis, Phillip Scott

**Affiliations:** 1 Department of Pathobiology, School of Veterinary Medicine, University of Pennsylvania, Philadelphia, PA, 19104, United States of America; 2 Department of Dermatology, Perelman School of Medicine, University of Pennsylvania, Philadelphia, PA, 19104, United States of America; 3 Serviço de Imunologia, Universidade Federal da Bahia, Salvador, Bahia, Brazil; 4 Instituto Nacional de Ciências e Tecnologia—Doenças Tropicais, Salvador, Bahia, Brazil; 5 Instituto de Ciências da Saúde, Universidade Federal da Bahia, Salvador, Bahia, Brazil; 6 Department of Microbiology, Perelman School of Medicine, University of Pennsylvania, Philadelphia, PA, 19104, United States of America; INRS - Institut Armand Frappier, CANADA

## Abstract

Cutaneous leishmaniasis is a disease characterized by ulcerating skin lesions, the resolution of which requires an effective, but regulated, immune response that limits parasite growth without causing permanent tissue damage. While mechanisms that control the parasites have been well studied, the factors regulating immunopathologic responses are less well understood. IL-22, a member of the IL-10 family of cytokines, can contribute to wound healing, but in other instances promotes pathology. Here we investigated the role of IL-22 during leishmania infection, and found that IL-22 limits leishmania-induced pathology when a certain threshold of damage is induced by a high dose of parasites. *Il22* ^*-/-*^ mice developed more severe disease than wild-type mice, with significantly more pathology at the site of infection, and in some cases permanent loss of tissue. The increased inflammation was not due to an increased parasite burden, but rather was associated with the loss of a wound healing phenotype in keratinocytes. Taken together, these studies demonstrate that during cutaneous leishmaniasis, IL-22 can play a previously unappreciated role in controlling leishmania-induced immunopathology.

## Introduction

Cutaneous leishmaniasis is a major neglected tropical disease affecting about 12 million people globally [1]. The spectrum of clinical manifestations in cutaneous leishmaniasis ranges from self-limiting nodules to non-healing ulcers with a highly inflammatory immune response, and the disease is caused by several different species of leishmania that reside within phagocytic cells. Control of the parasites requires IFN-γ produced by CD4+ Th1 cells [2]. However in spite of a Th1 response, some patients exhibit severe non-healing lesions [3,4]. Thus, in addition to controlling the parasites, regulating the inflammatory response is essential for disease control. TNF-α [5,6], IL-1β [7,8] and IL-17 [9,10] have all been implicated in promoting pathology in leishmaniasis, and damage caused by cytolytic CD8 T cells can also contribute to these immunopathologic responses[11–14]. IL-10 can regulate some of these immunopathologic responses [10,15]. Since drug treatment is often ineffective [5], and no human vaccine exists for the disease, a better understanding of the factors that mediate lesion resolution is essential to help develop new immunotherapies for the disease.

Recently, members of the IL-10 subfamily have been identified as key players in the wound healing process [16–18]. IL-22 is a prominent member of this family, and can instruct non-immune cells, such as epithelial cells and fibroblasts, to proliferate, migrate, and mend the extracellular matrix after injury [19,20]. These functions are important in maintaining surface barrier integrity and protection against subsequent infections. Additionally, IL-22 has been shown to induce the production of antimicrobial peptides from epithelial cells in order to maintain a balanced commensal population and prevent dysbiosis [21–23]. However, while IL-22 is important for tissue protection and contributes to wound healing in the skin, gut, and lungs [20,24,25], it can also be pathogenic in other inflammatory conditions, such as psoriasis [26]. These pathologic responses are mediated by some of the same functions of IL-22 that are protective, including uncontrolled proliferation and the production of inflammatory molecules [26–29]. Why IL-22 is protective in some situations and pathologic in others is unclear, but may depend on the amount of IL-22 produced, as well as the presence of other inflammatory cytokines such as IL-17 [29,30].

Like in some patients, the lesions of C57BL/6 mice normally heal after *L*. *major* infection. In order to determine if IL-22 contributes to resolution of a leishmanial infection, we infected *Il22* ^*-/-*^ mice with *Leishmania major* and *L*. *braziliensis* and monitored the course of infection. We found that *Il22* ^*-/-*^ mice exhibited increased tissue pathology compared with infections in wild-type mice. The absence of IL-22 did not influence the parasite burden, but rather led to higher levels of keratin 6a and keratin 16, both of which have been implicated in inhibiting the wound healing capabilities of keratinocytes [31,32]. We discovered that a role for IL-22 was only evident with high doses of parasites, suggesting that a threshold of inflammation might have to be reached before IL-22 contributed to tissue protection. Taken together, our results demonstrate a previously unknown role for IL-22 in limiting pathology during leishmania infection.

## Materials and Methods

### Ethics statement

This study was conducted according to the principles specified in the Declaration of Helsinki and under local ethical guidelines (Ethical Committee of the Maternidade Climerio de Oliveira, Salvador, Bahia, Brazil; and the University of Pennsylvania Institutional Review Board). This study was approved by the Ethical Committee of the Federal University of Bahia (Salvador, Bahia, Brazil) (010/10) and the University of Pennsylvania IRB (Philadelphia, PA) (813390). All patients provided written informed consent for the collection of samples and subsequent analysis. This study was carried out in strict accordance with the recommendations in the Guide for the Care and Use of Laboratory Animals of the National Institutes of Health. The protocol was approved by the Institutional Animal Care and Use Committee, University of Pennsylvania Animal Welfare Assurance Number A3079-01.

### Mice

Female C57BL/6 mice 6–8 weeks old were purchased from the National Cancer Institute (Frederick, MD). B6.IL22 (*Il22* ^*-/-*^) were donated by Pfizer (Cambridge, MA). All mice were maintained in specific pathogen-free facilities at the University of Pennsylvania. Prior to infection, mice were anesthetized using a ketamine and xylazine mixture and monitored until mice were fully awake. At the end of the experiments, mice were humanely euthanized using carbon dioxide inhalation. All procedures were performed in accordance with the guidelines of the University of Pennsylvania Institutional Animal Care and Use Committee (IACUC).

### Parasite and infection

*L*. *major* (WHO /MHOM/IL/80/Friedlin wild-type *L*. *major*) and *L*. *braziliensis* (MHOM/BR/01/BA788) [33] promastigotes were grown to the stationary phase in Schneider’s Drosophila medium (GIBCO BRL, Grand Island, NY, USA) supplemented with 20% heat-inactivated fetal bovine serum (FBS, Invitrogen USA), 2 mM l-glutamine, 100 U of penicillin and 100 μg of streptomycin per mL. Infective-stage promastigotes (metacyclics) were isolated from 4–5 day old (*L*. *major*) and 7 day old (*L*. *braziliensis*) stationary culture by density gradient separation by Ficoll (Sigma) [34]. Mice were inoculated intradermally in the ear with 10 uL of PBS containing 2 x 10^6^ *L*. *major* metacyclic promastigotes. In some experiments mice were infected with a low does of parasite (2 x 10^3^) or a super-high dose of parasites (2 x 10^7^). Lesion development was measured weekly by ear thickness with a digital caliper (Fisher Scientific). Mice were also assessed for pathology, using the following score system: no lesion (0), swelling/redness (1), deformation of the ear pinna (2), ulceration (3), partial tissue loss (4), and total tissue loss (5). Parasite burden in lesion tissues was assessed using a limiting dilution assay as previously described [35]. Freeze-thawed antigen (FTAg) was obtained from stationary-phase promastigotes of *L*. *major*. Soluble leishmanial antigen (SLA) was prepared from *L*. *braziliensis* parasites are previously described[36].

### Patients and recall assays

All cutaneous leishmaniasis patients were seen at the health post in Corte de Pedra, Bahia, Brazil, which is a well-known area of *L*. *braziliensis* transmission. The criteria for diagnosis were a clinical picture characteristic of cutaneous leishmaniasis in conjunction with parasite isolation or a positive delayed-type hypersensitivity response to *Leishmania* antigen, plus histological features of cutaneous leishmaniasis. In all cases, the immunological analysis was performed before therapy. For cell culture and IL-22 measurement, peripheral blood mononuclear cells (PBMCs) were obtained from heparinized venous blood layered over a Ficoll-Hypaque gradient (GE Healthcare), then washed and resuspended in RPMI1640 medium with 10% heat inactivated human AB serum (Sigma) at a concentration of 3 x 10^6^ cells/mL. These cells were added to 24-well plates and were kept unstimulated or were stimulated with soluble leishmania antigen (5 ug/mL) for 96 h at 37C in 5% CO2. The supernatants were collected and stored frozen until analyzed for cytokines. IL-22 was measured by enzyme-linked immunosorbent assay (Pfizer).

### Preparation of dermal sheets

The dorsal and ventral sides of the mouse ear were split mechanically and placed dermis side down in a 24 wells plate in RPMI 1640 containing 0.25 mg/mL of Liberase TL (Roche, Diagnostics Corp.) and 10 μg/mL DNase I (Sigma-Aldrich). Ears were incubated for 90 min at 37°C in a 24-well plate. Dermal cell suspensions were prepared by dissociation on 70- um cell strainer (Falcon) in PBS containing 0.05% BSA and 20 μM EDTA.

### *In vitro* restimulation and cytokine measurements

For measurements of antigen-specific cytokine production in the mouse, the retroauricular lymph node was removed, mechanically dissociated, and single cell suspensions were prepared. Cells were resuspended in RPMI 1640 supplemented with 10% of FBS, 2 mM l-glutamine, 100 U of penicillin and 100 μg of streptomycin per mL and 0.05 μM of β-mercaptoethanol. 4 x10^6^ cells per mL were plated in 24-well plates. Cells were incubated at 37°C in 5% CO2 with 20 x10^6^ *L*.*major* or *L*. *braziliensis* FTAg/mL. Supernatants were harvested 72 h after stimulation and assayed using a sandwich enzyme-linked immunosorbent assay (ELISA) for IFN-γ (eBioscience), IL-17 (eBioscience), and IL-22 (Pfizer). Cytokine concentrations were calculated from standard curves with a detection limit of 0.030 ng/mL.

### Antibodies and flow cytometry

Single cell suspensions from the ear were obtained as described above. For analysis of surface markers and intracellular cytokines, some cells were incubated for 4 h with 10 μg/mL of brefeldin A, 50 ng/mL of PMA and 500 ng/mL ionomycin (Sigma-Aldrich). Before staining, cells were incubated with an anti-Fcγ III/II receptor and 10% rat-IgG in PBS containing 0.1% BSA. Cells were stained for dead cells (Invitrogen) and surface markers (CD4, CD8β [BioLegend], CD45, Ly6G, CD11b [eBioscience]) followed by fixation with 2% of formaldehyde. The data were collected using LSRII flow cytometer (BD) and analyzed using FlowJo software (Tree Star).

### RNA isolation, purification, and quantitative real-time PCR

Total RNA was extracted from ear tissue samples in 700uL of RLT lysis buffer (Qiagen). The sample was homogenized using a tissue homogenizer (FastPrep-24, MP Biomedical), and total RNA was extracted according to the recommendations of the manufacturer and further purified using the RNeasy Mini kit (QIAGEN). RNA was reverse transcribed using high capacity cDNA Reverse Transcription (Applied Biosystems). Real-time RT-PCR was performed on a ViiA 7 Real-Time PCR System (Applied Biosystems). Relative quantities of mRNA for several genes was determined using SYBR Green PCR Master Mix (Applied Biosystems) and by the comparative threshold cycle method, as described by the manufacturer. mRNA levels for each sample were normalized to Ribosomal protein S11 gene (RPS11). Primers were designed using Primer Express software (version 2.0; Applied Biosystems); *Rps11*, forward, 5’-CGTGACGAAGATGAAGATGC-3’ and reverse, 5’-GCACATTGAATCGCACAGTC-3’; Krt5, forward, 5'-TTTGCCTCCTTCATCGACA-3' and reverse, 5'-CGGATCCAGGTTCTGCTTTA-3'; *Krt14*, forward, 5'-ATCGAGGACCTGAAGAGCAA-3' and reverse, 5'-TCGATCTGCAGGAGGACATT-3'; *Krt6a*, forward, 5'-GAGGAGAGGGAGCAGATCAA-3' and reverse, 5'-CACTTGGTGTCCAGGACCTT-3'; *Krt16*, forward, 5'-TTGAGGACCTGAAGAGCAAGA-3' and reverse, 5'-CCTGGCATTGTCAATCTGC-3'; *Il22*, 5'-ATGAGTTTTTCCCTTATGGGGAC-3' and reverse, 5'-GCTGGAAGTTGGACACCTCAA-3'; *Il22bp*, forward, 5'-TCAGCAGCAAAGACAGAAGAAAC-3' and reverse, 5'-GTGTCTCCAGCCCAACTCTCA-3'; *Ifng*, forward, 5'-GACTGTGATTGCGGGGTTGT-3' and reverse, 5'-GGCCCGGAGTGTAGACATCT-3'; *Il4*, forward, 5'-ATGGAGCTGCAGAGACTCTT-3' and reverse, 5'-AAAGCATGGTGGCTCAGTAC-3'; *Il17*, forward, 5'-CATGAGTCCAGGGAGAGCTT-3' and reverse, 5'-GCTGAGCTTTGAGGGATGAT-3'; *Il12p40*, forward, 5'-TTGAAAGGCTGGGTATCGGT-3' and reverse, 5'-GAATTTCTGTGTGGCACTGG-3', *Tnfa*, forward, 5'-TCACTGGAGCCTCGAATGTC-3' and reverse, 5'-GTGAGGAAGGCTGTGCATTG-3'; *Il6*, forward, 5’-ACAGAAGGAGTGGCTAAGGA-3’ and reverse, 5’-CACCATGGAGCAGCTCAG- 3’; *Il10*, forward, 5'-TGTCCAGCTGGTCCTTTGTT-3’ and reverse, 5'-ACTGCACCCACTTCCCAGT-3'; *Tgfb*, forward, 5’-CGCTGCTACTGCAAGTCAGA-3’ and reverse, 5’-GGTAGCGATCGAGTGTCCA-3’; *Il27p28*, forward, 5'-GATTGCCAGGAGTGAACCTG-3' and reverse, 5'-CGAGGAAGCAGAGTCTCTCAG-3'; *Il1a*, forward, 5’-TTGGTTAAATGACCTGCAACA-3’ and reverse, 5’-GAGCGCTCACGAACAGTTG-3’; *Il1b*, forward, 5’-TTGACGGACCCCAAAAGAT-3’ and reverse, 5’- GATGTGCTGCTGCGAGATT-3’.

### Microbiome collection, sequencing, and analysis

Two independent experiments were performed using littermates as controls, with n = 9–10 mice per cohort for a total of *9 Il22* ^*-/-*^ mice, *3 Il22* ^*+/-*^ mice, and *7 Il22* ^*+/+*^ mice. Microbiota was collected from the ear of the mouse using a swab (Catch-all Sample Collection Swab, Epicentre) moistened in Yeast Cell Lysis Buffer (from MasterPure Yeast DNA Purification Kit; Epicentre). DNA was isolated from swab specimens and amplification of the 16S-V4 region was performed as previously described [37]. Sequencing of 16S rRNA amplicons was performed at the Penn Next Generation Sequencing Core using the Illumina MiSeq platform with 150 bp paired-end ‘V2’ chemistry.

### Pre-processing and community characterization of 16S rRNA sequence data

Sequence pre-processing followed methods previously described [37], but modified by subsampling at 11,000 sequences per sample. QIIME 1.6.0[38] was used for initial stages of sequence analysis. Sequences were clustered into OTUs (operational taxonomic units, a proxy for ‘species’) using UCLUST [39] at 97% sequence similarity. Bacterial diversity was calculated using the following alpha diversity indices: 1) Shannon diversity index; 2) Faith’s phylogenetic distance (PD); and 3) Chao I species estimation; and 4) number of observed OTUs. Relative abundance of bacteria was calculated based on taxonomic classification of sequences using the RDP classifier [40] at a confidence threshold of 0.8. Microbiome data was analyzed with the R statistical software environment (www.r-project.org). Statistical significance was determined using two-sample Wilcoxon tests and corrected for multiple comparisons by FDR where appropriate.

### Statistical analysis

Results represent means ± SEM. Data were analyzed using Prism 5.0 (GraphPad Software, San Diego, CA). Statistical significance was determined by one-way ANOVA when comparing more than two groups and by an unpaired two-tailed Student’s t test to compare means of lesion sizes, parasite burdens, and cytokine production from different groups of mice. Statistically significant differences were defined as * when *p* values <0.05.

## Results

### Leishmania infections induce the production of IL-22

**Fig 1:**
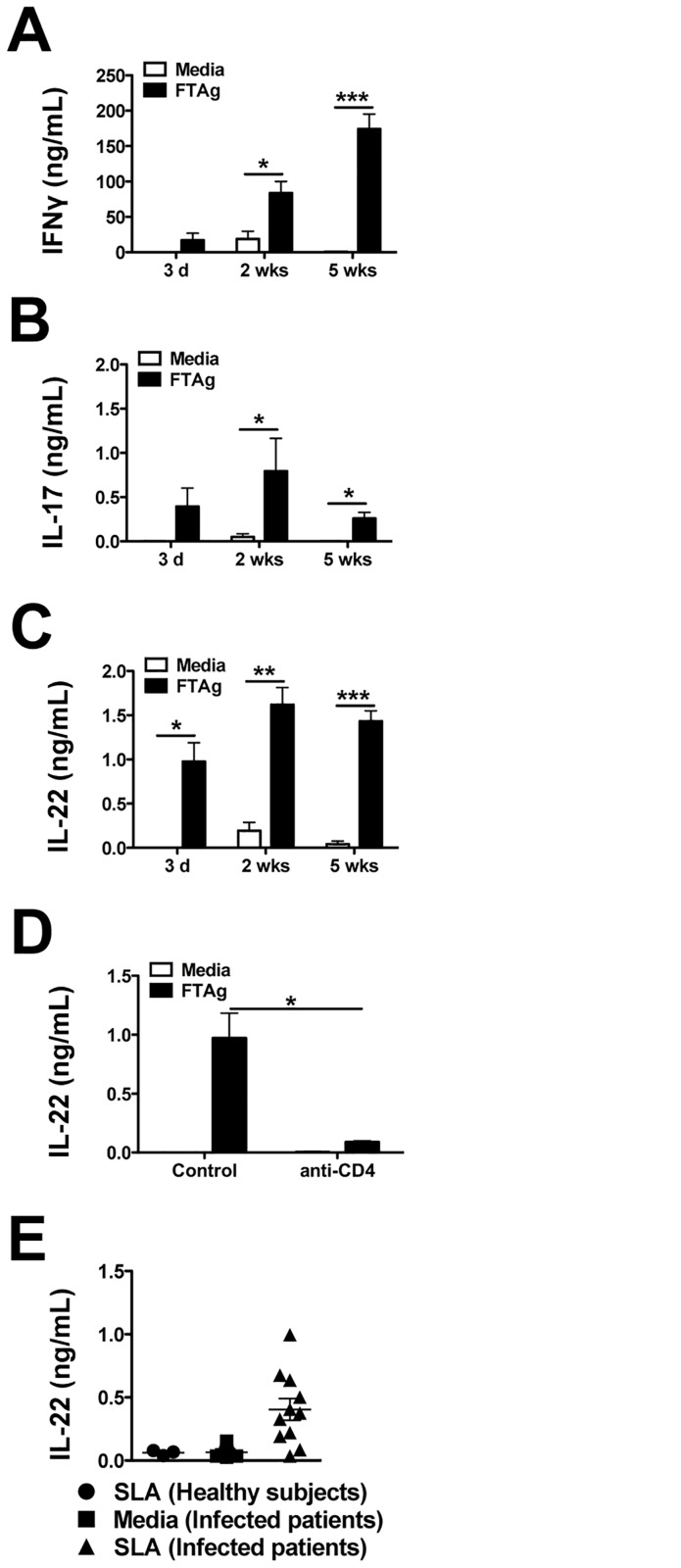
IL-22 is induced during leishmania infections. C57BL/6 mice were intradermally infected with 2 x 10^6^ *L*. *major* promastigotes metacyclics in the ear. Cells from the draining lymph nodes of infected mice were isolated and cultured for 72 hours with media or leishmania antigen. Supernatants were collected and **(A)** IFN-γ **(B)** IL-17, and **(C)** IL-22 release was measured by ELISA. **(D)** C57BL/6 mice were intradermally infected with 2 x 10^6^ *L*. *major* promastigotes metacyclics in the ear and two days later treated with anti-CD4. Mice were euthanized on day 3 and cells from the draining lymph nodes were isolated and cultured for 72 hours with media or leishmania antigen to analyze IL-22 production by ELISA. **(E)** PBMCs from healthy subjects and *L*. *braziliensis* infected patients were cultured for 72 hours with media or *L*. *braziliensis* antigen. Supernatants were collected and analyzed for IL-22 release by ELISA. Data are representative of at least 3 independent experiments, with 3–5 mice per group. Error bars indicate mean ± SEM, *p < 0.05, **p < 0.01, ***p < 0.001.

Since IL-22 can have tissue protective effects, we investigated whether IL-22 might help control pathology during infection with leishmania. We first asked whether infection with leishmania parasites led to an increase in IL-22 production. C57BL/6 (wild-type) mice were infected with *L*. *major* and were euthanized at 3 days, 2 weeks or 5 weeks after infection. Cells from the draining lymph nodes were stimulated with leishmanial antigen and cytokine levels were assessed. As expected during infection with *L*. *major*, IFN-γ and IL-17 were produced in an antigen dependent manner (Fig 1A and 1B). As early 3 days after infection there was an antigen specific production of IL-22, which was maintained at 2 and 5 weeks post-infection (Fig 1C). Because we know CD4+ T cells can be a major source of IL-22 [27,41], we wanted to determine if CD4+ cells contributed to the antigen-specific production of IL-22 during *L*. *major* infection. Thus, C57BL/6 mice were infected with *L*. *major* and depleted of CD4+ cells in vivo using a neutralizing antibody 2 days prior to sacrificing the mice. Cells were harvested from the draining lymph nodes at 3 days post-infection and cultured with media alone or with *L*. *major* antigen for 72 hours. Antigen stimulated cells from anti-CD4 treated mice produced significantly less IL-22 than untreated mice (Fig 1D), demonstrating that the production of IL-22 is dependent on the presence CD4+ T cells. We also observed the production of IL-22 from cells of mice infected with another species of the parasite, *L*. *braziliensis* (data not shown). To determine if patients infected with *L*. *braziliensis* parasites also produced IL-22, peripheral blood mononuclear cells (PBMCs) from leishmaniasis patients were isolated and cultured with leishmanial antigen. Similar to cells from mice, PBMCs from infected patients, but not healthy subjects, produced IL-22 in response to stimulation with leishmanial antigen (Fig 1E), suggesting that IL-22 may be important in human patients as well as in experimental murine infections.

### IL-22 limits pathology during leishmania infection independent of parasite control

**Fig 2:**
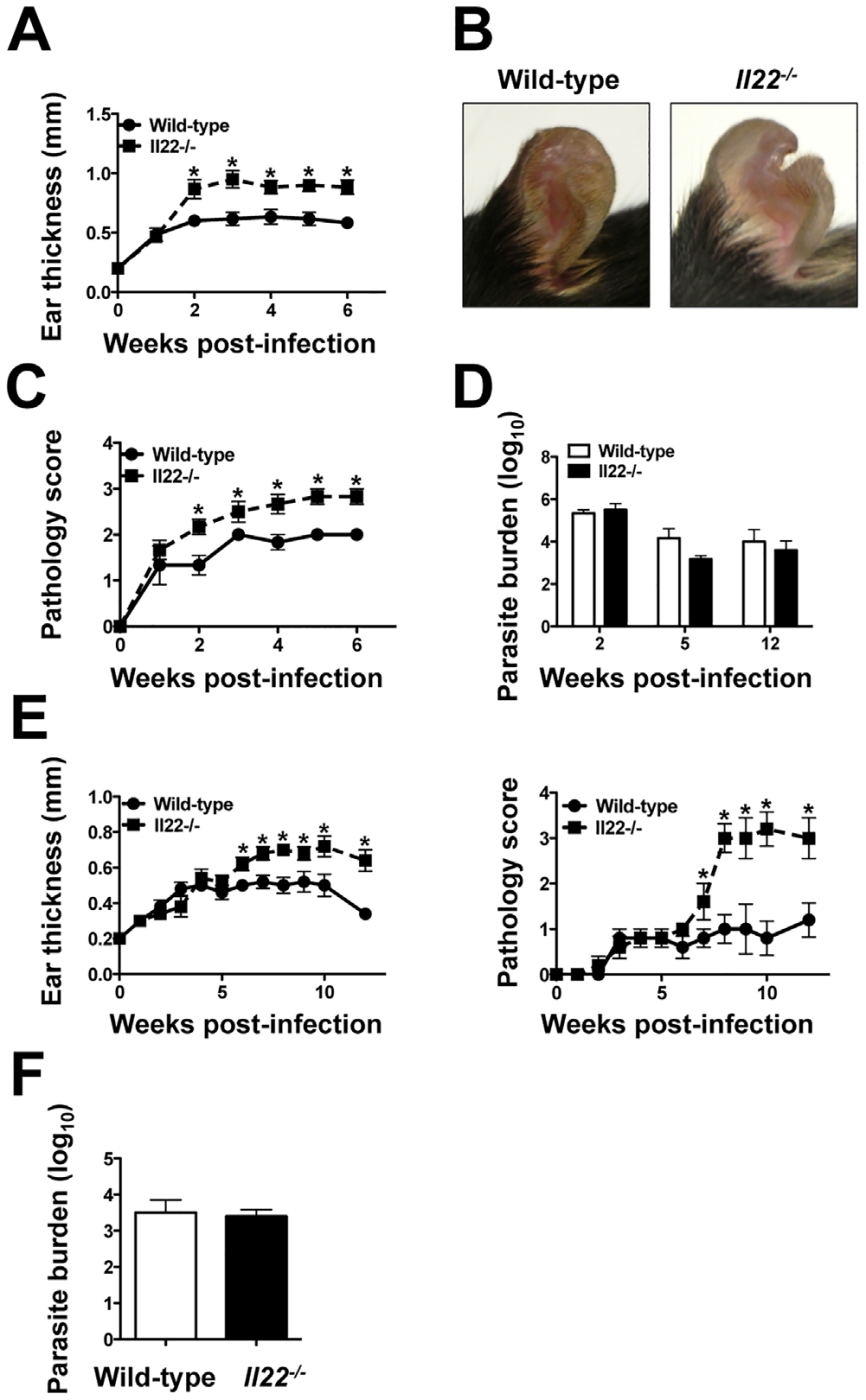
IL-22 limits pathology during leishmania infection. **(A)** C57BL/6 (wild-type) and *Il22* ^*-/-*^ mice were intradermally infected with 2 x 10^6^ *L*. *major* promastigote metacyclics and euthanized at various time-points after infection. The lesions were assessed by measuring ear thickness for 6 weeks. **(B)** Pictures were taken at 5 weeks post-infection. **(C)** Lesion pathology was determined based on a pathology score. **(D)** Number of parasites in the lesions was quantified using a limiting assay at 2, 5, and 12 weeks post-infection. **(E)** Wild-type and *Il22* ^*-/-*^ mice were intradermally infected with 2 x 10^6^ *L*. *braziliensis* promastigote metacyclics and lesions were assessed by measuring ear thickness and given a pathology score for 12 weeks and **(F)** parasite numbers were quantified using a limiting dilution assay in the lesions at 12 weeks post-infection. Data are representative of at least 2 independent experiments, with 3–5 mice per group. Error bars indicate mean ± SEM, *p < 0.05.

To determine if IL-22 plays a protective role during the course of infection with leishmania, C57BL/6 and *Il22* ^*-/-*^ mice were infected with *L*. *major* and the disease monitored. *Il22* ^*-/-*^ mice exhibited larger lesions compared with wild-type mice (Fig 2A). We noticed that in addition to greater swelling, the ears of *Il22* ^*-/-*^ mice often exhibited more severe pathology than wild-type mice, and in some cases led to tissue loss at the site of infection (Fig 2B). To quantify these changes, we employed a scoring system that better captures the pathology associated with leishmania infection. As seen in Fig 2C, *Il22* ^*-/-*^ mice exhibited greater pathology than wild-type mice infected with *L*. *major*. To determine if the increased pathology observed following *L*. *major* infections was due to higher parasite levels in *Il22* ^*-/-*^ mice, we assessed the parasite burden in wild-type and *Il22* ^*-/-*^ mice at 2, 5 and 12 weeks of infection, and found no significant differences (Fig 2D).

*L*. *braziliensis* parasites are known to induce a particularly strong inflammatory response in patients, and also cause mucosal leishmaniasis, the most severe form of the disease [42]. Therefore, we asked if IL-22 regulated the lesion resolution in this infection as well. We infected wild-type and *Il22* ^*-/-*^ mice with *L*. *braziliensis* and followed the course of infection. As with *L*. *major*, *L*. *braziliensis* infected *Il22* ^*-/-*^ mice had significantly larger lesions than wild-type mice with more pathology, but no differences in the number of parasites within the lesions (Fig 2E and 2F).

### IL-22 maintains wound-healing capabilities in the skin during *L*. *major* infection

**Fig 3:**
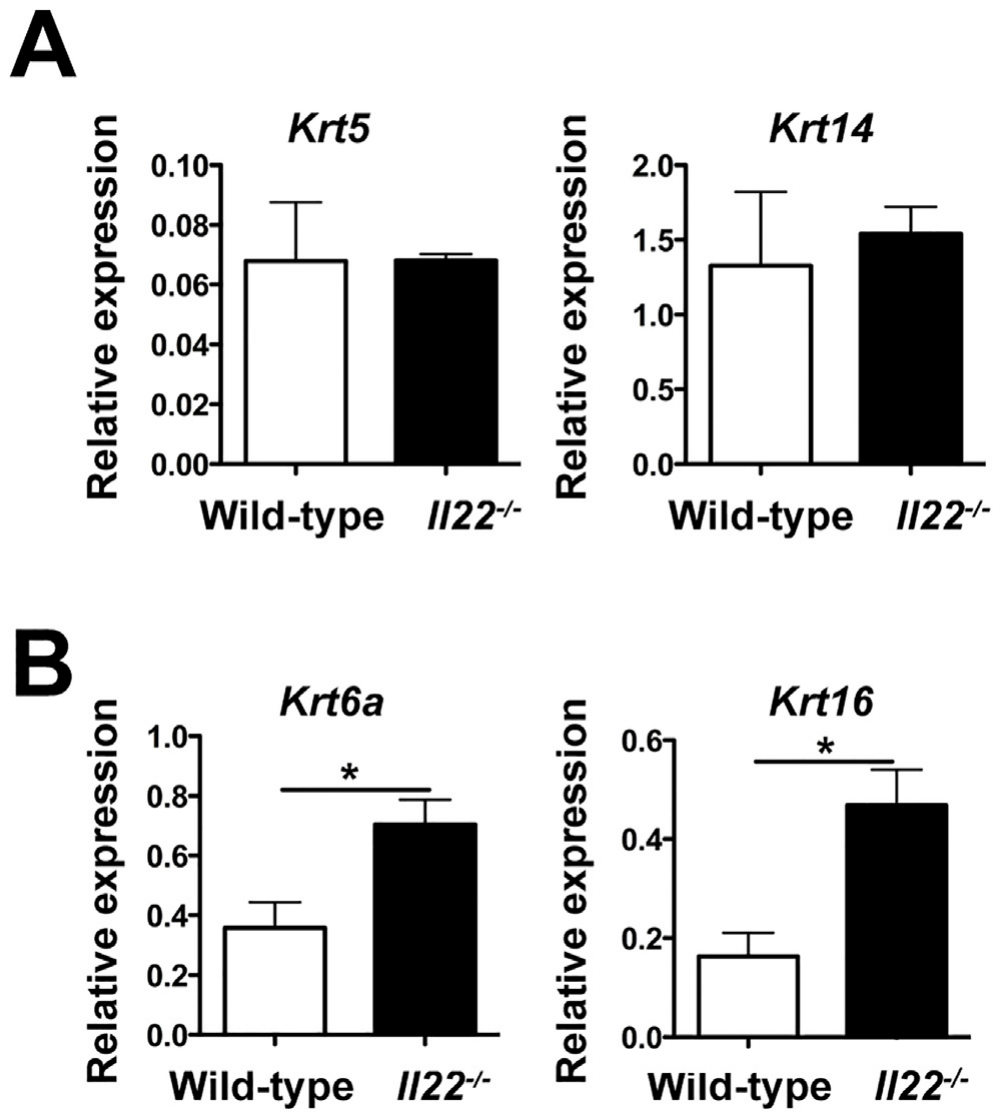
IL-22 regulates the expression of skin repair genes during *L*. *major* infection. **(A-B)** Wild-type and *Il22* ^-/-^ mice were intradermally infected with 2 x10^6^ *L*. *major* promastigote metacyclics and RNA was isolated from the lesions at 5 weeks post-infection to assess gene expression. Data are represented as relative expression to housekeeping gene *rps11* and are representative of at least 2 independent experiments, with 3–5 mice per group. Error bars indicate mean ± SEM, *p < 0.05.

The resolution of a leishmanial lesion is analogous to wound healing, which requires keratinocyte proliferation and differentiation [43]. Therefore, we analyzed the expression of several genes at the peak of infection to assess keratinocyte functions in the lesions of wild-type and *Il22* ^*-/-*^ mice. We observed no difference in the expression of keratin 5 and keratin 14, both of which are expressed in proliferating keratinocytes, between wild-type and *Il22* ^*-/-*^ mice (Fig 3A). We then decided to look at other keratins which are upregulated in chronic wounds and can inhibit the ability of keratinocytes to efficiently heal wounds and damage [31,32]. We observed that *Il22* ^*-/-*^ mice had higher expression of keratin 6a and keratin 16 (Fig 3B), both of which are known to inhibit keratinocytes migration. Thus, one role of IL-22 during cutaneous leishmaniasis may be to promote wound healing capabilities of keratinocytes by regulating the expression of keratins involved in migration and differentiation.

### The requirement for IL-22 depends on parasite burden and inflammation

**Fig 4:**
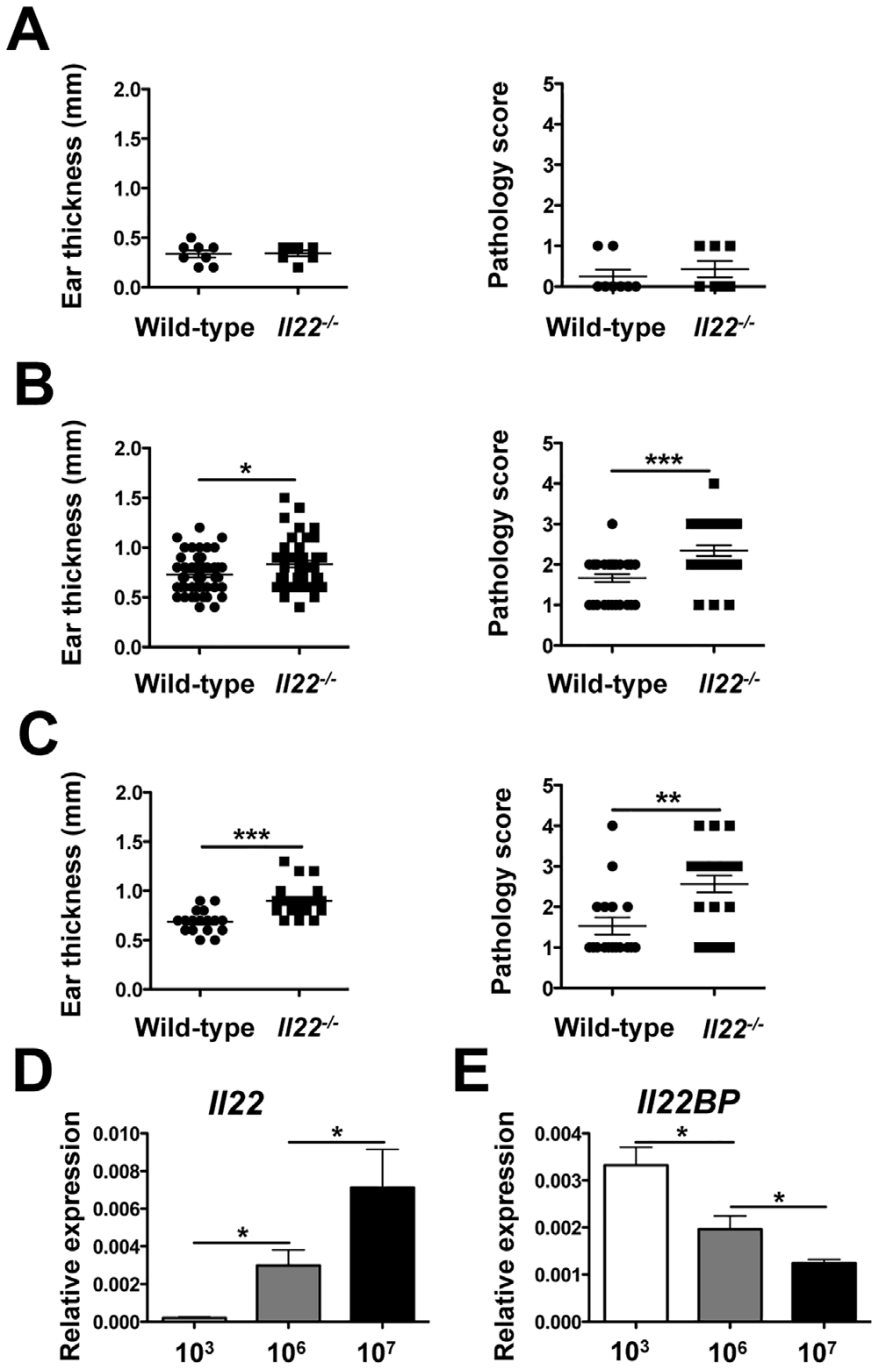
The requirement for IL-22 is parasite dose dependent. Lesion sizes and pathology scores were compiled from several experiments at 5 weeks post-infection from wild-type and *Il22* ^*-/-*^ mice that were intradermally infected with **(A)** 2 x 10^3^, **(B)** 2 x 10^6^, or **(C)** 2 x 10^7^ *L*. *major* metacyclics. RNA was isolated from the lesions of wild-type mice infected with *L*. *major* to assess **(D)** *Il22* and **(E)** *Il22BP* expression. Data are represented as relative expression to housekeeping gene *rps11* and are representative of at least 2 independent experiments, with 3–5 mice per group. Error bars indicate mean ± SEM, *p < 0.05,*p < 0.01, ***p < 0.001.

Recently, it was reported that IL-22 does not play a role during a low dose of infection with *L*. *major* [44]. Our results, taken together with other findings prompted us to consider the possibility that IL-22 might only be required when a threshold of inflammation and tissue damage was present. To test this hypothesis, we infected mice with a super high dose of parasites (2 x 10^7^), an intermediate dose (2 x 10^6^), and with a low dose of parasites (2 x 10^3^), and followed the course of infection. Because we noticed some variability between experiments, we decided to pool data from multiple experiments and compare pathology at the peak of infection. Similar to recent findings in which mice were infected with a low dose of parasites [44], we observed no difference in the lesion size or pathology between wild-type and *Il22* ^*-/-*^ mice when infected with 2 x 10^3^ parasites (Fig 4A). On the other hand, *Il22* ^*-/-*^ mice infected with 2 x 10^6^ and 2 x 10^7^ parasites had more pathology than their wild-type counterparts (Fig 4B and 4C). We euthanized these animals at 5 weeks post-infection and assessed their parasite burdens. As expected from the results described above, no differences were observed in the parasite burden between wild-type and *Il22* ^*-/-*^ mice (data not shown). We then measured levels of IL-22 expression in the lesions, and found significantly higher expression *of Il22* mRNA when mice were infected with more parasites (Fig 4D). The IL-22 binding protein (IL-22BP) is a soluble receptor that inhibits IL-22 signaling through its receptors [45]. Thus, we examined the expression of *Il22BP* in wild-type mice infected with *L*. *major* infection. Unlike IL-22, IL-22BP was expressed at significantly lower levels when mice were infected with more parasites (Fig 4E). These results suggest that following infection with high numbers of parasites IL-22 is induced to a greater extent and less inhibited by IL-22BP, and that IL-22 helps regulate the pathology associated with a higher parasite burden.

### IL-22 does not modulate the skin microbiome at the steady state

**Fig 5:**
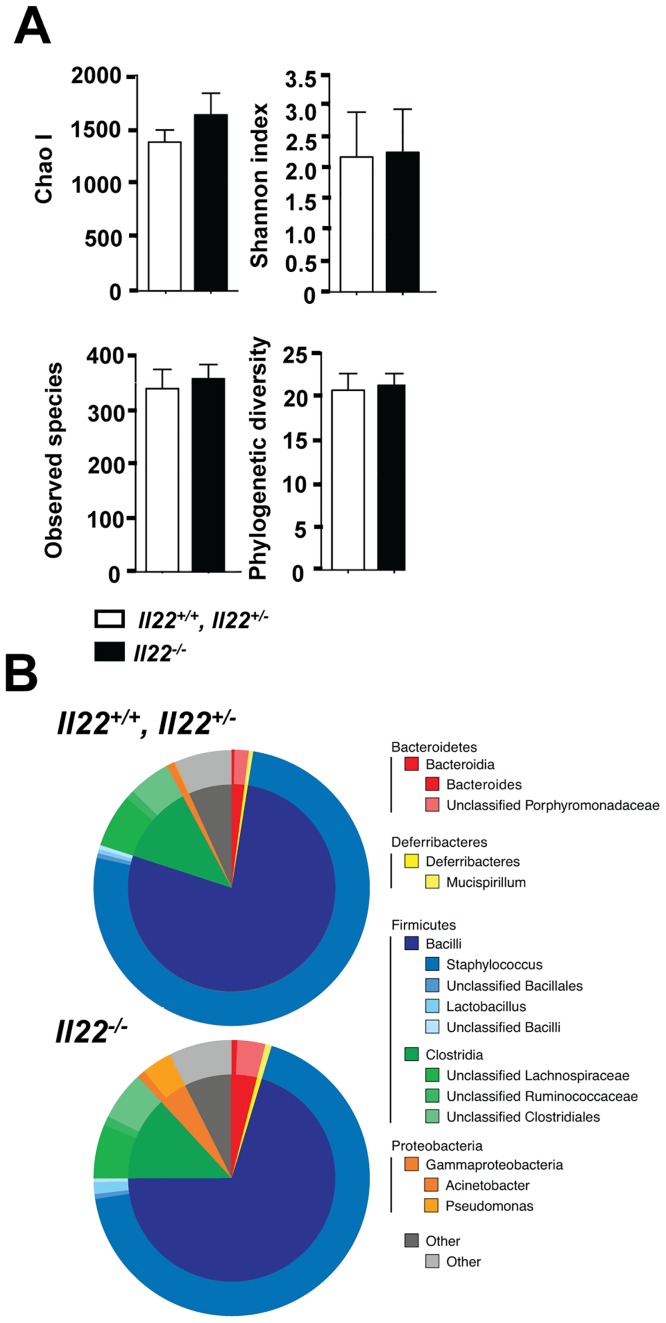
IL-22 does not modulate the skin microbiome at the steady state. Swabs were collected from *Il22* ^*+/+*^, *Il22* ^*+/-*^, and *Il22* ^*-/-*^ cohoused littermates and bacterial DNA was isolated and sequenced. **(A)** Within sample diversity was calculated using four commonly utilized alpha metrics: Chao I, Shannon Index, Observed Species, and Faith’s Phylogenetic Diversity. **(B)** The microbiome composition was calculated at multiple phylogenetic levels. The outer ring represents the relative contributions of the 12 most prevalent genera. The inner ring represents the corresponding class for each genus. The remaining genera were compiled into the “Other” category depicted in gray. Data are representative of 2 independent experiments, with 4–5 mice per group.

Recent studies indicate that the skin microbiome influences the pathology associated with leishmania infection [46]. Since IL-22 regulates the production of antimicrobial peptides (AMPs) [41,47], we considered the possibility that homeostatic levels of IL-22 might influence AMP levels, resulting in changes in the skin microbiome and consequently disease development. To test this idea, the ears of uninfected *Il22* ^*+/+*^/*Il22* ^*+/-*^ and *Il22* ^*-/-*^ littermates were swabbed to extract bacterial DNA. 16S ribosomal RNA genomic sequencing was performed and the skin microbiome was analyzed. In two independent experiments (n = 9 *Il22* ^*-/-*^ mice and n = 10 control littermate mice) no significant differences in bacterial diversity were observed between littermate controls and *Il22* ^*-/-*^ mice (Fig 5A). There were also no differences in the relative abundance of the bacterial communities between controls and *Il22* ^*-/-*^ mice (Fig 5B). These findings indicate that *Il22* ^*-/-*^ mice do not have a dysbiotic skin microbiome responsible for the increased pathology.

### IL-22 does not regulate inflammatory cell infiltrate, but rather limits tissue damage during *L*. *major* infection

**Fig 6:**
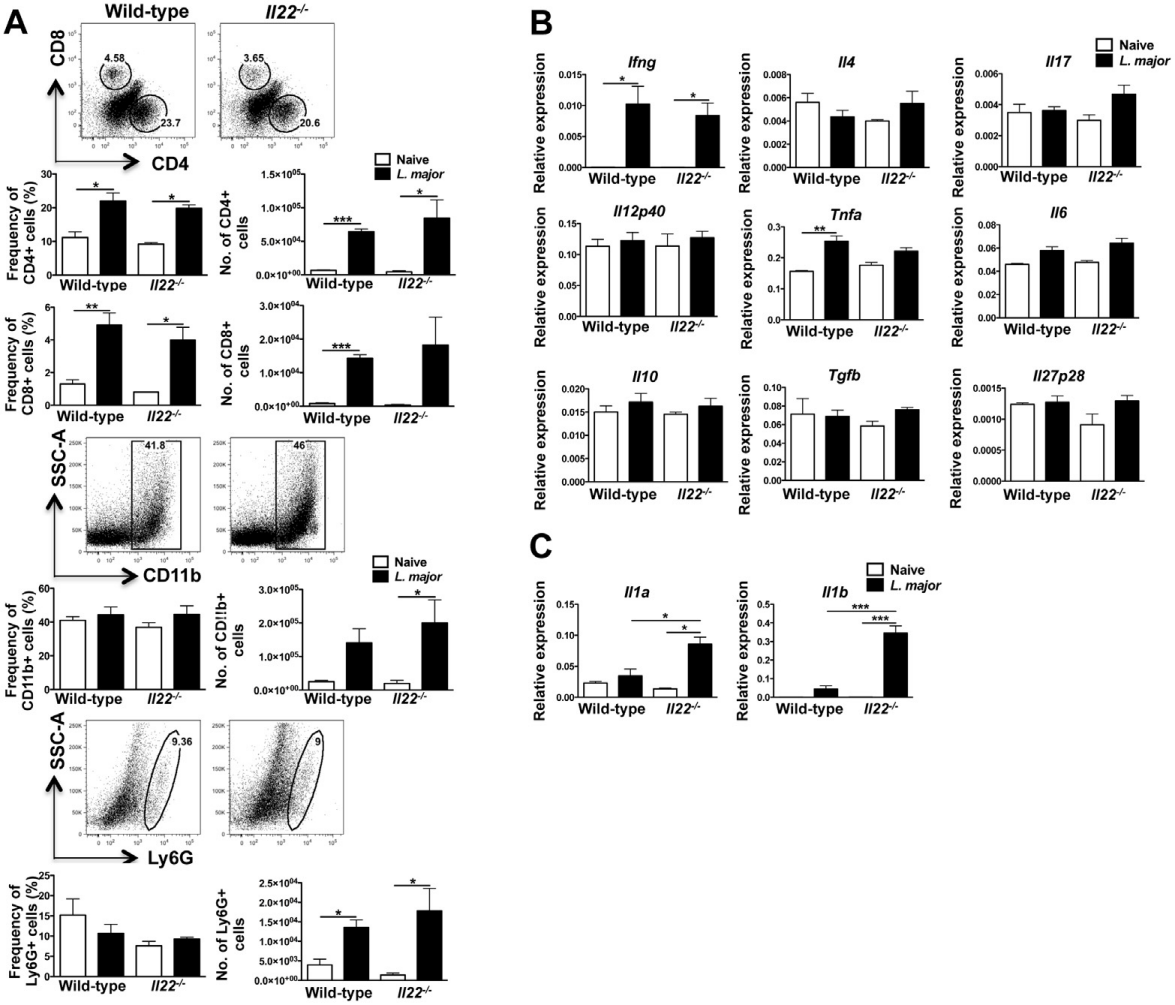
IL-22 does not alter the immune response during *L*. *major* infection. Wild-type and *Il22* ^-/-^ mice were intradermally infected with 2 x10^6^ *L*. *major* promastigote metacyclics and cells from 5 week old lesions were collected and analyzed by flow cytometry. **(A)** Representative dot plots and bar graphs depict frequencies and total cell numbers of CD4+, CD8+, CD11b+, and LY6G+ cells. **(B-C)** RNA was isolated from the lesions of wild-type mice infected with *L*. *major* to assess gene expression. Data are represented as relative expression to housekeeping gene *rps11* and are representative of at least 3 independent experiments, with 3–5 mice per group. Error bars indicate mean ± SEM, *p < 0.05,*p < 0.01, ***p < 0.001.

Because we observed more pathology and inflammation in the *Il22* ^*-/-*^ mice, we wanted to determine if there was increased inflammatory cell infiltrate in the lesions of these mice. We examined the presence of CD4+ and CD8+ T cells, CD11b+ myeloid cells, and neutrophils in the lesions of *L*. *major* infected wild-type and *Il22* ^*-/-*^ mice at the peak of infection. While there was an increase over naïve skin in the frequency and numbers of T cells of wild-type *and Il22* ^*-/-*^ lesions and in the numbers of myeloid cells, there was no difference in these populations between wild-type and *Il22* ^*-/-*^ mice (Fig 6A). We also assessed transcript levels of inflammatory and regulatory cytokines in the lesions of wild-type and *Il22* ^*-/-*^ mice. As expected, *Ifng* levels were increased following infection, and there was a similar increase in wild-type and *Il22* ^*-/-*^ mice. There were minimal or no changes in *Il4*, *Il17*, *Tnfa*, *Il12a*, *Il6*, *Il10*, *Tgfb* and *Il27p28* gene expression between naïve skin and leishmanial lesions, and no significant differences between wild-type and *Il22* ^*-/-*^ mice (Fig 6B). However, we found that the lesions of *Il22* ^*-/-*^ mice had higher expression of *Il1a* and *Il1b* compared with wild-type mice (Fig 6C). The expression of these molecules is often observed in inflamed tissue and can be induced and released when cells encounter tissue damage [48]. Although there were no differences in the immune response between wild-type and *Il22* ^*-/-*^ mice, increased expression of these damage-associated molecules demonstrates that *Il22* ^*-/-*^ mice directly or indirectly regulate their production during infection with *L*. *major*.

## Discussion

Our results uncover a previously unknown role for IL-22 during cutaneous leishmaniasis. While a pathologic and inflammatory role for IL-22 has been reported in other cutaneous diseases [26,28,49], we found that IL-22 does not promote increased inflammation during infection with *Leishmania spp*. Rather, *Il22* ^*-/-*^ mice exhibited more tissue damage than wild-type mice when infected with *L*. *major* or *L*. *braziliensis*, suggesting that IL-22 limits pathology when a threshold of inflammation is reached during leishmaniasis.

Our results demonstrate that the production of IL-22 is dependent on the presence of CD4+ T cells, which have previously been shown to produce IL-22 [27,41]. However, γδ T cells, NK cells, ILCs and neutrophils are other potential sources of IL-22 that might contribute to the IL-22 observed in these lesions [26,50–52]. Interestingly, the production of IL-22 appeared to be dose-dependent, such that mice infected with higher doses of *L*. *major* expressed higher levels of IL-22 in the lesions. Inflammation and damage in other models of disease have been shown to induce IL-22 expression [21,27,29,53,54], consistent with our findings that higher doses of *L*. *major* elicit more inflammation and higher expression of IL-22. Conversely, we observed a decrease in the expression of the IL-22 antagonist, IL-22BP, in mice with higher doses of the parasite. This inverse relationship of IL-22/IL-22BP regulating tissue damage has also been observed during Hepatitis C and schistosome infections [55]. Thus, we hypothesize that having a high IL-22/IL-22BP ratio is required to limit pathology.

In order to determine whether the immune response was influenced by the absence of IL-22, we assessed cytokine responses within leishmanial lesions of *Il22* ^*-/-*^ mice. Changes in the balance of Th1 and Th2 cytokines is often associated with increased susceptibility to *L*. *major*, but since there were no differences in the parasite burden it was not surprising that the mRNA levels of *Ifng*, *Tnfa*, *Il12p40 and Il4* were similar in both wild-type and *Il22* ^*-/-*^ mice. Moreover, there were no differences in the cellular infiltrate of T cells and myeloid cells in the lesions of wild-type and *Il22* ^*-/-*^ mice. These results prompted us to consider other ways in which IL-22 can provide tissue protection during inflammation.

*L*. *major* infection leads to the development of ulcerated lesions that eventually resolve due to tissue remodeling at the infection site [56–58]. IL-22 promotes wound healing by increasing epithelial cell proliferation, decreasing the differentiation of keratinocytes and inducing anti-apoptotic molecules in keratinocytes [19,59–61]. Thus, one way IL-22 may enhance wound healing in leishmaniasis is by regulating *L*. *major* induced keratinocyte death. Additionally, IL-22 stimulates fibroblasts to produce extracellular matrix proteins, as well as increases the differentiation of myofibroblasts that help to contract wounds [20], and both of these functions could be critical in the resolution of leishmanial lesions. In this study, we found another mechanism in which IL-22 contributes to wound healing and tissue repair. Keratinocyte proliferation and differentiation are critically regulated processes during wound repair [43]. Upon injury, activated keratinocytes migrate to close the wound, while basal keratinocytes proliferate at the basement membrane [62,63]. In order for a cell to proliferate and repair the basement membrane, differentiation must be halted [62]. IL-22 can induce proliferation, but also down-regulate keratinocyte differentiation and keratin expression [19]. Thus, we decided to examine the expression of various proliferation and differentiation markers. While the proliferation markers, keratin 5 and keratin 14 were unaffected by the absence of IL-22, the lesions of *Il22* ^*-/-*^ mice expressed higher levels of keratins 6a and 16. These genes are induced in keratinocytes upon injury and are maintained during reepithiliazation. However, the intensity in expression levels of these keratins is important because their overexpression can lead to defects in keratinocyte migration and wound closure [31]. The higher expression of keratins 6a and 16 observed in chronic wounds is consistent with our data showing that *Il22* ^*-/-*^ mice have a defect in wound repair during *L*. *major* infection. Interestingly, lower expression of keratin 16 or deletion of keratin 6a can enhance keratinocyte migration [32], which may explain the eventual lesion resolution in wild-type mice with lower expression of these keratins. Keratinocyte differentiation and migration are key to wound healing, and thus our results suggest that IL-22 may be important in regulating these processes through keratins 6a and 16 in order to efficiently resolve leishmanial lesions.

While IL-22 protects against certain pathogens, such as *Klebsiella pneumonia* and *Citrobacter rodentium* [21,25], in our study we found no evidence that IL-22 contributes to control of *L*. *major* or *L*. *braziliensis*, as wild-type and *Il22* ^*-/-*^ mice contained the same number of parasites in their lesions. These results are similar to those observed with other parasites, such as toxoplasma or schistosomes [64]. However, this is in contrast to visceral leishmaniasis, where the production of IL-22 has been correlated with increased protection [65,66]. How IL-22 promotes resistance in visceral leishmaniasis is unknown, but it is unlikely to be a direct effect on the parasites, since the IL-22R is not expressed on the cells infected with leishmania [67]. Since stromal cells play a role in immunoregulation in visceral leishmaniasis, one possibility is that stimulation of stromal cells by IL-22 might indirectly influence the development of disease [68].

IL-22 helps maintain barrier function in the skin, but when produced at high levels and/or in the context of other proinflammatory cytokines, such as IL-17, IL-22 promotes increased pathology [29]. The factors that determine whether IL-22 will play a protective or pathologic role remain poorly understood, although it has been suggested that the nature of the inflammatory response may be a determining factor [29]. Our results indicate that one factor determining whether IL-22 is important in protection in the skin may be the degree of damage induced. Thus, when *Il22* ^*-/-*^ mice were infected with a high dose of parasites, we routinely saw increased pathology in *Il22* ^*-/-*^ mice compared with wild-type mice, while we found no differences in the development of lesions in *Il22* ^*-/-*^ mice and wild-type mice when the animals were infected with a low dose of parasites. The latter finding would account for the results of a prior study where IL-22 was reported to have no role in *L*. *major* infection [44]. These results suggest that the protective role for IL-22 requires a threshold of inflammation that is reached at high parasite doses in this experimental model. This raises the issue of how our murine studies relate to human leishmaniasis. While the initial dose of parasites transmitted by sandflies is much less than the high doses we have studied here, patients also exhibit significantly more pathology than what occurs in low dose infections in mice. Thus, we hypothesize that in more severe forms of cutaneous leishmaniasis, as often seen following *L*. *braziliensis* infection, IL-22 might be induced to ensure that even more severe disease does not develop. Consistent with this was our finding that cells from patients made IL-22 in response to stimulation, indicating that there was sufficient damage in the patients to promote IL-22 production.

Taken together, our results in *Il22* ^*-/-*^ mice show that IL-22 limits pathology during cutaneous leishmaniasis and suggest that once a certain threshold of damage is reached, IL-22 is expressed at higher levels and limits subsequent damage by maintaining skin barrier integrity and wound healing capacities. In the absence of IL-22, not only do lesions fail to resolve, but higher expression of the inflammatory molecules IL-1α and IL-1β may lead to even greater tissue destruction. Thus, IL-22 plays an important, and previously unappreciated, role in maintaining skin repair properties and limiting inflammation during cutaneous leishmanial infections.

## Supporting Information

S1 ARRIVE Guidelines Checklist The Animal Research: Reporting of *In Vivo* Experiments (ARRIVE) checklist outlines where information can be found regarding the suggested guidelines. (PDF)
